# Supplementary material for: CuS-131I-PEG Nanotheranostics-Induced “Multiple Mild-Hyperthermia” Strategy to Overcome Radio-Resistance in Lung Cancer Brachytherapy
Source: Pharmaceutics. 2022 Nov 30;14(12):2669. doi: 10.3390/pharmaceutics14122669 (PMC9785376; doi:10.3390/pharmaceutics14122669)
Supplement: Supplementary file 1 [file pharmaceutics-14-02669-s001.zip › pharmaceutics-2012950-supplementary.pdf]

CuS-^{131}I-PEG nanotheranostics-induced “multiple mild-hyperthermia” strategy to overcome radio-resistance in lung cancer brachytherapy

Yanna Cui [1,†], Hui Yan [1,†], Haoze Wang [1,2], Yongming Zhang [1], Meng Li [1,2], Kai Cui [1], Zeyu Xiao [1,*], Liu Liu [1,*], Wenhui Xie [1,*]

1 Department of Nuclear Medicine, Shanghai Chest Hospital & Department of Pharmacology and Chemical Biology, Translational Medicine Collaborative Innovation Center, Shanghai Jiao Tong University School of Medicine, Shanghai 200025, China.

2 College of Chemistry and Materials Science, Shanghai Normal University, Shanghai 200233, China.

* Correspondence: zxiao@sjtu.edu.cn (Z. X.); chestnuclear@126.com (L. L.); xknuclear@163.com (X. W.)

† These authors contributed equally to this work.

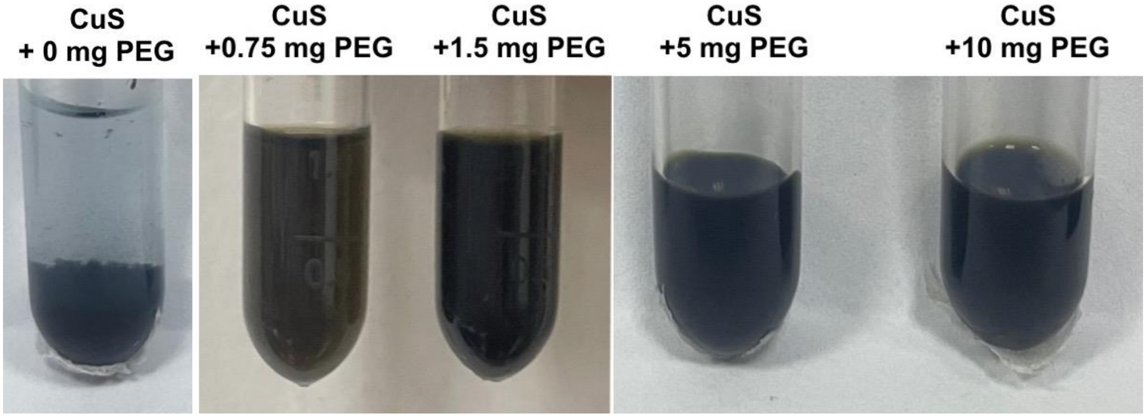

Figure S1. Images of CuS NPs without and with PEG modification.

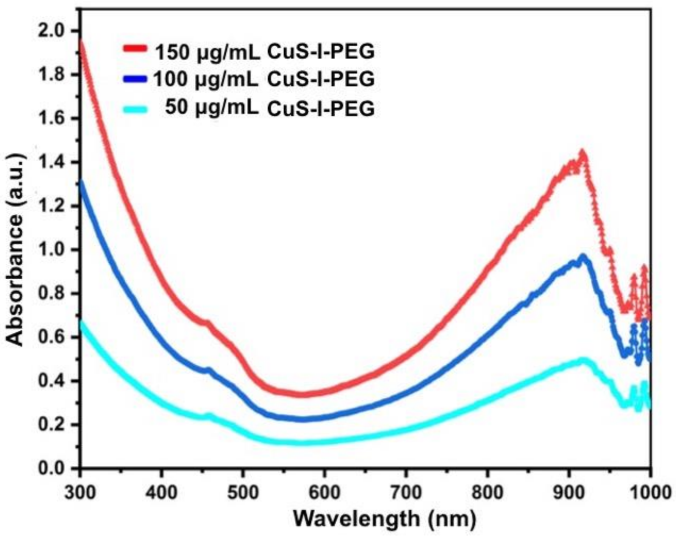

Figure S2. UV-Vis-NIR absorption spectra of CuS-I-PEG nanotheranostics with different concentraiton of CuS, i. e. 150 μg/mL, 100 μg/mL and 50 μg/mL, respectively.

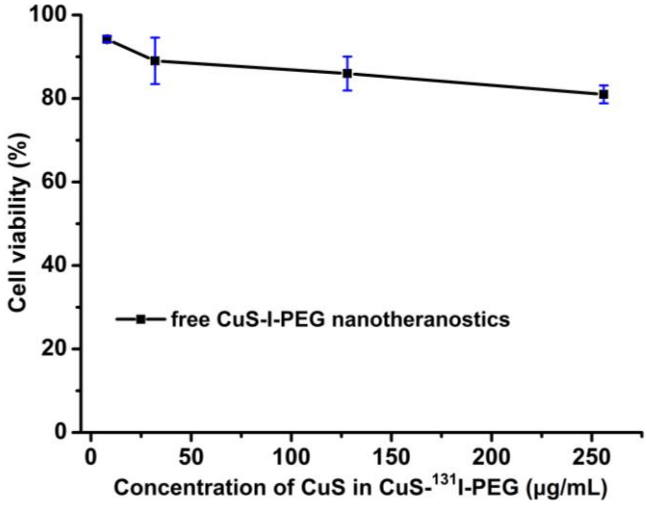

Figure S3. Cytotoxicity of free CuS-I-PEG nanotheranostics after incubation with A549 cells for 24 h.

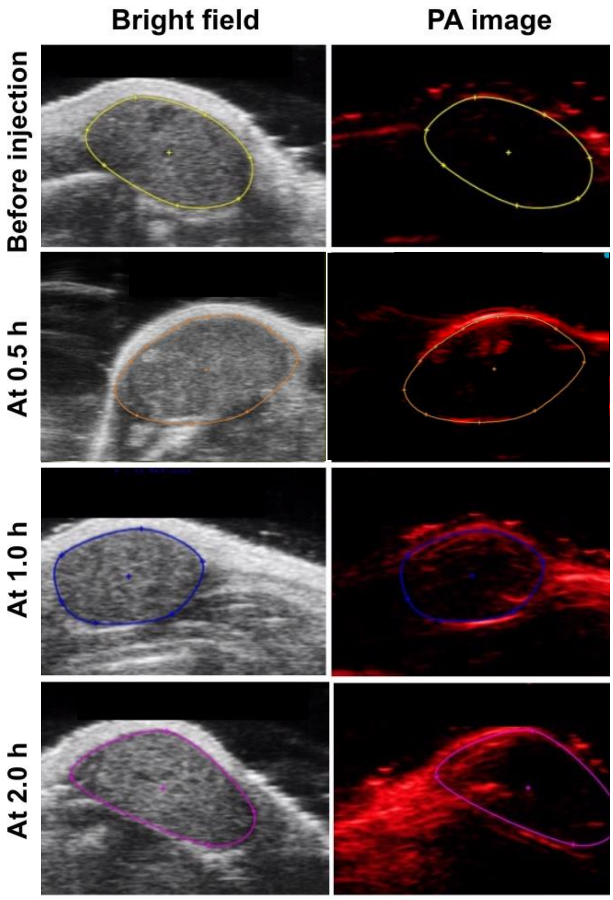

Figure S4. Photoacoustic images of CuS-^{131}I-PEG nanotheranostics at tumors without 808 nm NIR laser irradiation.

Table S1. Formulations with different amount of CuS, iodine and PEG, and their size and zeta potential results.

Formulations	CuS(mg)	NaI(mg)	CuS:NaI(mol ratio)	SH-PEG_{5K}(mg)	Size(Number mean d.nm)	Zeta potential
1	3.3625	/	/	0	10	-13
2	3.3625	/	/	0.5	10	-20
3	3.3625	/	/	0.75	15	-20
4	3.3625	/	/	1.5	17	-21
5	3.3625	/	/	2.5	18	-21
6	3.3625	/	/	5	16	-37
7	3.3625	/	/	10	20	-32
8	3.3625	0	0	1.5	15	-14
9	3.3625	0.1875	20:1	1.5	18	-20
10	3.3625	0.25	15:1	1.5	19	-14
11	3.3625	0.375	10:1	1.5	18	-14
12	3.3625	0.75	5:1	1.5	15	-13
13	3.3625	0.375	10:1	0.5	16	-15
14	3.3625	0.375	10:1	0.75	22	-20
15	3.3625	0.375	10:1	1.5	19	-16
16	3.3625	0.375	10:1	2.5	20	-19
